# Progress in Control-Actuation Robotic System for Gastrointestinal NOTES Development

**DOI:** 10.1155/2022/7047481

**Published:** 2022-10-30

**Authors:** Huijiang Du, Xiaotong Liu, Hudie Sun, Qi Zhu, Liping Sun

**Affiliations:** ^1^School of Medical Instruments, Shanghai University of Medicine and Health Sciences, Shanghai, China; ^2^Sino-European School of Technology, Shanghai University, Shanghai, China

## Abstract

**Purpose:**

Natural orifice transluminal endoscopic surgery (NOTES) is a minimally invasive surgical procedure that reduces patient trauma, infection probability, and rehabilitation time. This paper reviews the progress made in the control-actuation robotic systems for gastrointestinal NOTES development. *Material and Methods*. A survey on both existing and state-of-the-art control-actuation robotic systems for gastrointestinal NOTES was conducted in December 2021.

**Results:**

Nine control-actuation robotic systems for gastrointestinal NOTES were identified. The structures and specifications of these robotic systems were reported. The technical parameters were also discussed. Special attention was directed to systems using a control-actuation structure and tendon-driven mechanism. The control-actuation robotic systems typically deploy a control-actuation structure and tendon-driven mechanism. Control-actuation robotic systems for gastrointestinal NOTES show great ability to improve operational accuracy and flexibility and flatten the learning curve of procedures. These characteristics suggest that the use of control-actuation robotic systems is worth exploring in future development.

## 1. Introduction

High-quality intra-abdominal surgery cannot be realized if flexible endoscopes alone are used, because it requires appropriate countertraction and accuracy of tasks. To overcome such problems, some robotic systems have been developed [1]. These systems have shown their value for NOTES [2]. NOTES can reduce patient trauma, the probability of infection, and rehabilitation time and can lead to improved cosmesis by minimizing external incisions [3–6]. Nevertheless, manipulating tissue using long and slender endoscopic instruments is challenging [7]. The ability to introduce instruments into body cavities, triangulate instruments, and apply force on tissue is limited by the endoscopic approach [8]. Key technologies such as the control-actuation structure and tendon-driven mechanism have been proposed [9]. This paper provides an overview of control-actuation structure robotic systems for gastrointestinal NOTES in ascending order of publication year.

## 2. Overall Design

The design of a robotic system for gastrointestinal NOTES typically includes a master console and two mechanical arms. The end of the mechanical arm can be a gripper or an electrosurgical unit (ESU). As per the author's knowledge, in all the reported designs, the endoscope and mechanical arms are placed in an overtube. By decreasing the diameter of the mechanical arm and optimizing the design, the diameter of the overtube is decreased as much as possible to reduce patient discomfort and increase patient tolerance. A smaller overtube diameter also makes it easier to pass through narrow and curved natural orifices during the procedure [10].

The performance of the robotic system for gastrointestinal NOTES is mainly to measure the flexibility of the distal part of the mechanical arm, gripping force [10], and diameter of the overtube. The flexibility of the distal part of the mechanical arm refers to the degrees of freedom (DOF). The gripping force is the maximum effort exerted by the end effector (gripper), which is normally expressed as a force unit (Newton (N)).

## 3. Control-Actuation Structure

**Figure 1:**
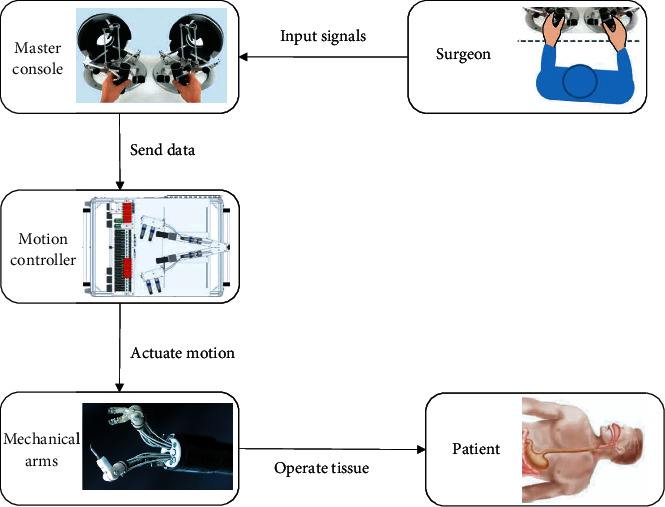
The architecture of the control-actuation structure [15, 16].

The robotic system for gastrointestinal NOTES typically adopts a control-actuation structure. The structure includes three parts: a master console, a motion controller, and mechanical actuation arms, which are connected in series. The master console collects the control actions of the surgeon, converts them into angular data, and sends the angular data to the motion controller. Manipulators are used as master console devices, such as joysticks or Geomagic Touch. The motion controller interprets the angular data and sends the results to the mechanical arms [11]. The mechanical arms actuate the corresponding motions [12]. In the actuation process, the antidither algorithm can effectively filter the dithering of the hand and make the actuation more precise and stable [13]. Therefore, robotically controlled actuation of mechanical arms can provide high precision [14].  Figure 1 illustrates the architecture of the control-actuation structure.

## 4. Tendon-Driven Mechanism

**Figure 2:**
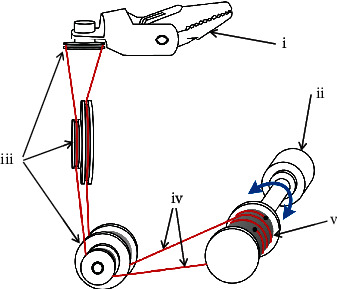
Tendon-driven mechanism [1]: (i) end effector, (ii) motor, (iii) pulley, (iv) tendon, and (v) driving drum.

The tendon-driven mechanism imitates animal ligaments and muscles. The mechanical actuation arms and end effectors are driven by the pulling of the tendon, which is driven by servomotors [17]. The tendons are arranged along the axis of the mechanical actuation arm. The mechanical arm is manipulated to bend or distort by pulling different tendons. Pulling the tendon which connects the gripper in a certain direction opens it. Moreover, pulling the tendon in the opposite direction causes the gripper to close. A tendon-driven mechanism requires a plurality of servomotors and pulleys; therefore, the system is inevitably bulky [18]. The tendon-driven mechanism is relatively simple to implement compared with other driving schemes [19].  Figure 2 illustrates the tendon-driven mechanism.

## 5. Control-Actuation Robotic Systems for Gastrointestinal NOTES

**Figure 3:**
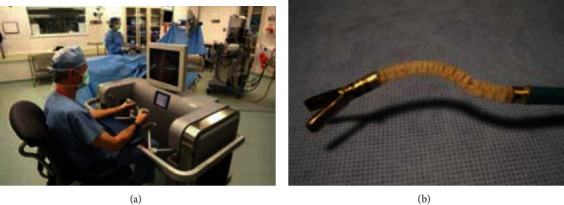
Master console and one of the mechanical actuation arms of the ViaCath system [7]: (a) master console and (b) mechanical actuation arm.

Abbott et al. developed the ViaCath system. It consists of a master console, two mechanical actuation arms, and a long flexible instrument. Both the endoscope and mechanical actuation arms enter the gastrointestinal tract through a 19 mm diameter overtube in parallel. The end effectors of the two mechanical actuation arms are located in front of the endoscope and can operate under camera monitoring. Each mechanical actuation arm has 8 DOF and a gripper. The gripping force of the gripper is 3 N. The system was validated in the excised stomach and in vivo porcine models [7].  Figure 3 shows the master console and one of the mechanical actuation arms of the ViaCath system.

**Figure 4:**
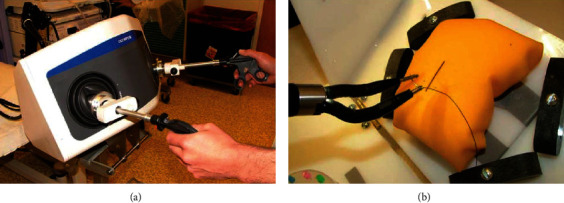
Master console and mechanical actuation arms of EndoSAMURAI™ [20]: (a) master console and (b) mechanical actuation arms.

EndoSAMURAI™ was developed by Olympus Corporation. The system includes a conventional endoscope, a master console, and two mechanical actuation arms in an 18 mm diameter overtube. The end effectors are biopsy forceps with serrated jaws and an ESU. Each mechanical actuation arm has 5 DOF. The value of the gripping force of the forceps is not specified. The two mechanical actuation arms are parallel and can be controlled using manipulators [20]. The system was validated in ex vivo and in vivo experiments [21, 22].  Figure 4 shows the master console and mechanical actuation arms of EndoSAMURAI™.

**Figure 5:**
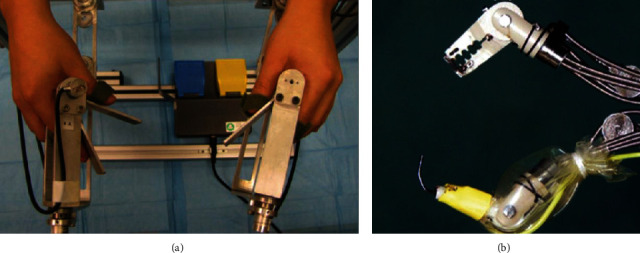
Master console and mechanical actuation arms of the EndoMaster [23]: (a) master console and (b) mechanical actuation arms.

Phee et al. and Ho et al. of the Nanyang Technological University in Singapore designed the EndoMaster system. The system adopts a control-actuation structure, which consists of a master console, a motion controller, and two mechanical actuation arms. The diameter of the overtube is 19.5 mm, and there are two channels with a diameter of 2.8 mm and 3.7 mm to pass through a gripper and an ESU. The master console responds to the surgeon's input; the motion controller controls the mechanical actuation arms, the gripper, and the ESU [23, 24]. The system was validated with animal experiments in vivo and then validated ESD in five patients [15, 25].  Figure 5 shows the master console and mechanical actuation arms of the EndoMaster.

**Figure 6:**
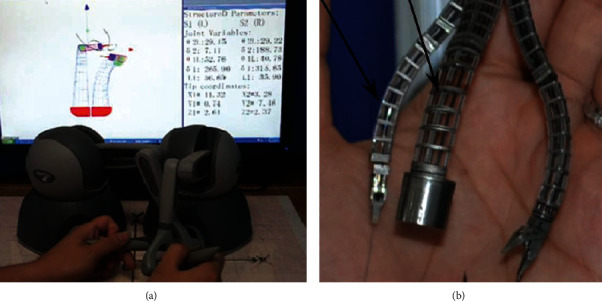
The mechanically and manually driven endoscopic testbed [26]: (a) master console and (b) mechanical actuation arms.

Zhao et al. developed a mechanically and manually driven endoscopic testbed. Two mechanical actuation arms and a master console are integrated into an overtube with a diameter of 12 mm. Each mechanical actuation arm is equipped with an end effector at the far end and has 5 DOF. However, the value of the gripping force was not specified. The testbed was validated by animal experiments in vitro [26].  Figure 6 shows the master console and mechanical actuation arms of the testbed.

**Figure 7:**
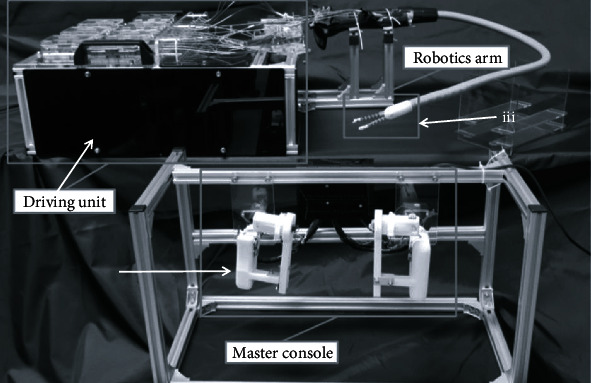
The structure of the two-armed control-actuation robot [27].

Lau et al. designed a two-armed robot for ESD. The robot adopts a control-actuation structure and the diameter of the overtube is 18 mm. One of the two mechanical actuation arms has a surgical gripper that could lift the mucosa at the far end. The other end effector is a unipolar ESU. The two arms pass through two 6 mm diameter instrument channels in the overtube, and each of them has 5 DOF. The gripping force of the gripper is 0.47 N. The robot was validated by animal experiments in vitro [27].  Figure 7 shows the structure of the robot.

**Figure 8:**
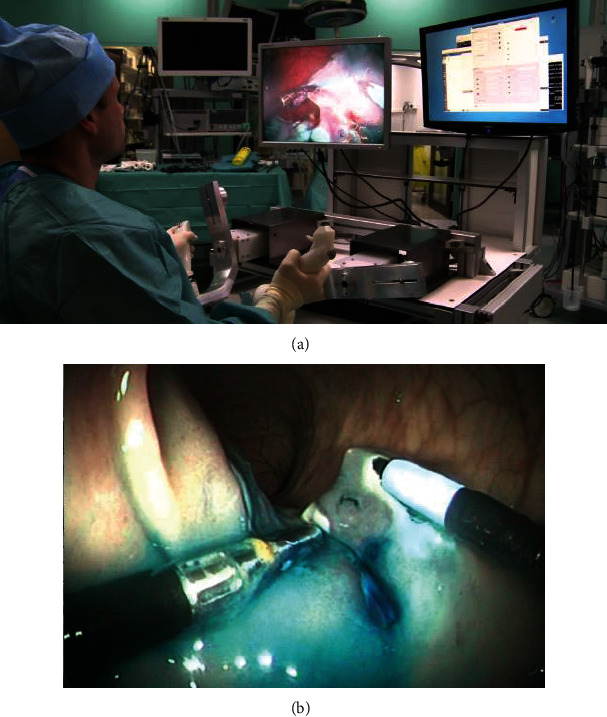
The STRAS system [28]: (a) master console and (b) the distal part of the STRAS system.

Zorn et al. designed a robotic system called the STRAS. The system consists of an ordinary endoscope, a master console, and two mechanical actuation arms. The diameter of the overtube is 16 mm, which allows it to house two 4.3 mm and one 3.2 mm diameter working channel for mechanical actuation arms. The overtube is equipped with a camera. The system has 10 DOF. The gripper could apply a force of 0.9 N. The STRAS was validated for ESD by animal experiments in vivo [28].  Figure 8 shows the master console and distal parts of the STRAS system.

**Figure 9:**
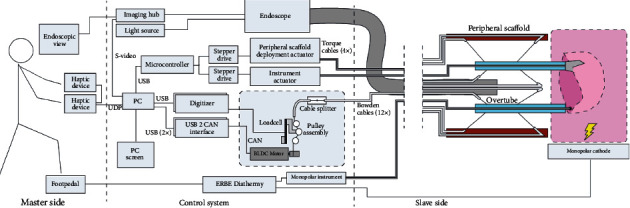
The CYCLOPS system [29].

Vrielink et al. released a gastrointestinal surgical robotic system called the CYCLOPS. The system adopts a control-actuation structure. The master console uses haptic devices with force feedback (Geomagic Touch) to indicate the workspace boundaries. A deployable scaffold exists at the end of the overtube. When the deployment is complete, the mechanical arms can move in all directions within the DOF range. The scaffold needs to enter the digestive tract from the cannula, and the maximum width of the scaffold in the folded state is 30 mm. Although the cannula diameter is not specified, it can be seen from the maximum width of the scaffold in the folded state that the diameter of the scaffold is greater than 30 mm. In the case of the bent overtube, the executive force of the end effector in the *X*-, *Y*-, and *Z*-axial directions changes from 3.47 N to 19.08 N. As of 2018, this system was prepared for clinical validation [29].  Figure 9 illustrates the CYCLOPS system.

**Figure 10:**
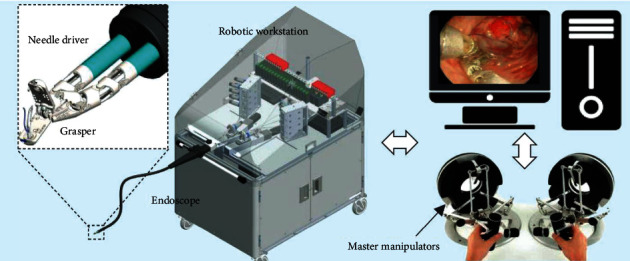
Overview of the EndoMaster EASE system [30].

EndoMaster EASE is a second-generation EndoMaster system. The system consists of master manipulators, actuation instruments, and a customized and commercialized endoscope [30]. No detailed information such as the diameter of the endoscope is specified. Two Omega7 haptic interfaces are used as master manipulators. The robotic endoscope is customized to have three instrument channels. One of the channels has a 2.5 mm diameter for the endoscopic tools to pass through, whereas the other two channels are where the 4.4 mm diameter needle driver and 4.2 mm diameter grasper can be smoothly inserted. The actuation instruments are interchangeable [30]. Each instrument has 5 DOF. And the gripping forces of the needle driver and grasper can be 4.3 and 5.8 N when the arms are straight [30]. More than 150 patients underwent ESD surgery using the EndoMaster EASE at the Wales Hospital in Singapore [31].  Figure 10 presents an overview of the EndoMaster EASE system.

**Figure 11:**
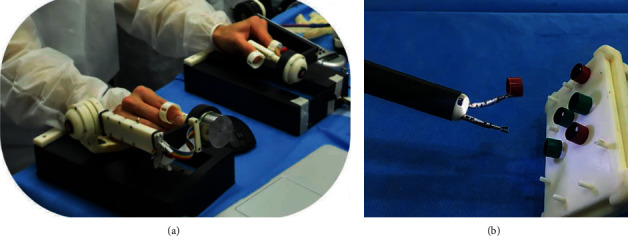
Overview of the K-FLEX system [32]: (a) master console and (b) mechanical actuation arms.

Hwang et al. proposed an endoscopic robotic platform called K-FLEX for intracavitary surgery. The system consists of a master console and two mechanical actuation arms. The robot has a total of 14 DOF and an overtube of 17 mm diameter. The joint structure of the mechanical actuation arm can accurately locate the movement. The gripping force of the gripper is 2.94 N. Ex vivo tests were validated in animal trials [32].  Figure 11 presents an overview of the K-FLEX system.

## 6. Discussion

The control-actuation robotic systems for gastrointestinal NOTES are listed in ascending order of publication year in Table 1.

This paper reported progress in the development of existing and state-of-the-art control-actuation robotic systems for gastrointestinal NOTES. All reported robotic systems have the configuration of a master console using manipulators and two mechanical actuation arms. The tendon-driven mechanism of mechanical actuation arms includes tendons, servomotors, and connecting rods. Each arm typically has 4–8 DOF to produce sufficient workspace and distal flexibility [32]. One of the end effectors is the gripper. The second type is usually an ESU.

Mechanical actuation arms must be introduced into the surgical site inside an overtube through a natural orifice. The gripping force of the gripper varies significantly in different designs owing to the influence of the diameter of the overtubes and mechanical actuation arms. The diameter of the overtubes ranges from 12 mm to 30 mm. A smaller diameter could easily be deployed through narrow and curved natural orifices during NOTES. However, the distal dexterity and gripping force of the mechanical arms are reduced [10]. From the perspective of both surgeons and patients, if the functional requirements of surgery such as dissection, suturing, and knot tying can be met, a smaller diameter is better than a larger diameter. Designers must balance minimizing the diameter of the overtube and maximizing the performance of the mechanical arm.

The filtering function of the motion controller eliminates the physiological tremor of the operator's hand, which allows operation with unprecedented accuracy [33]. The skills necessary to complete these operations require complex long-term practices. The control-actuation robotic system for gastrointestinal NOTES can flatten the learning curve of procedures and make it easier to perform complex operations in a standardized manner [16].

Force feedback can enable the surgeon to have a more natural interaction between surgical tools and tissue, as normally experienced during minimally invasive surgery [34]. Grippers with force-sensing functions based on the tactile sensing principle have been developed and tested with a sponge [35] and a laparoscopic training box [36]. Instruments specialized for certain operations in NOTES, such as suturing devices and grippers, will be developed [37].

The success of NOTES depends on the competence of the human surgeons and the degree of effectiveness of their coordination. One of the futuristic advancements is to replace the assistant surgeon with two robotic arms. To accomplish that, artificial intelligence- (AI-) based systems are required that not only can understand the complete surgical scene but also detect the actions being performed by the main surgeon in the current video frame. An AI algorithm based on image segmentation can be used to measure and warn gripper movements [38]. These advancements will lay the foundation for more robust algorithms which will be used in future surgical systems such as the autonomous assistant surgeon, surgeon feedback systems, and surgical anomaly detection [39].

Most reported systems are in the prototype testing stage or animal/human experimental stage [40]. We believe that with the growth in patient demand and technological progress, a control-actuation robotic system for gastrointestinal NOTES will be further developed.

## Figures and Tables

**Table 1:** Control-actuation robotic systems for gastrointestinal NOTES.

System	Diameter of overtube (mm)	DOF	Gripping force [N]	Research progress
ViaCath	19	(8 + 1) × 2	3	Animal in vivo
EndoSAMURAI™	18	5 × 2	—	Animal in vivo
EndoMaster	19.5	9	2.87	Patient trial
Testbed (Zhao)	12	(5 + 1) × 2	—	Animal in vitro
Robot (Lau)	18	10 + 2	0.47	Animal in vitro
STRAS	16	10 + 1	0.9	Animal in vitro
CYCLOPS	30	(5 + 1) × 2	19	Vivo preclinical
EndoMaster EASE	—	5 × 2	5.8	Patient trial
K-FLEX	17	14	2.94	Animal ex vivo

## Data Availability

The parameters and the pictures of the robotic systems used to support the finding of this study are included within the article.
